# Epidural Hematoma in Minor Hepatic Metastasectomy

**DOI:** 10.7759/cureus.59879

**Published:** 2024-05-08

**Authors:** Sofia Pereira, Sara Nunes, Mariana Luís

**Affiliations:** 1 Department of Anesthesiology, Hospital Central do Funchal, Funchal, PRT

**Keywords:** coagulation tests, hemostatic disorders, epidural catheter complication, liver resection, epidural hematoma

## Abstract

Liver resection poses many challenges for the anesthesiologist, including intraoperative hemodynamic instability, postoperative pain, and risk of coagulopathy. We report a case of epidural hematoma after epidural catheter removal, following a minor liver single metastasectomy. The main purpose of this case report is to bring to light the false security provided by traditional coagulation parameters and whether further investigation should be considered in selected cases, before handling neuraxial catheters. Alterations in coagulation after a partial hepatectomy remain poorly understood; thus, we believe that additional hemostatic values such as viscoelastic testing might be considered to better assess these patients.

## Introduction

Liver resection poses many challenges for the anesthesiologist, including intraoperative hemodynamic instability, postoperative pain, and the risk of coagulopathy [1].

Epidural anesthesia (EA) is often the preferred choice for pain relief following major abdominal surgery and has been recommended in the “Enhanced Recovery After Surgery” protocols [2]. However, hemostatic disorders frequently occur after hepatic resection, and, although extremely rare, epidural hematoma (EH) is one of the possible serious complications [2].

We report a case of EH after epidural catheter removal following a minor liver metastasectomy.

The main purpose of this case report is to bring to light the false security provided by traditional coagulation parameters, such as international normalized ratio (INR), and whether further investigation should be considered in selected cases, before handling neuraxial catheters.

Alterations in coagulation after a partial hepatectomy are not completely comprehended and remain difficult to predict [3]; thus, we believe that additional hemostatic parameters are needed to better assess patients. It has been advocated that thromboelastometry may provide valuable information concerning this matter [2].

This article was previously presented as a meeting abstract at the 2021 Euroanaesthesia Virtual Congress, from December 17 to 19, 2021.

## Case presentation

A patient in their 70s, diagnosed with metastatic cholangiocarcinoma and a past surgical history of cephalic pancreatoduodenectomy was admitted for an elective single metastasectomy. The lesion was minor (2x3 cm). Medical history involved hypertension medicated with bisoprolol and chemotherapy two years previously.

The preoperative assessment showed minor changes in coagulation profile and liver function tests, as described in Table 1.

**Table 1:** Perioperative coagulation and liver function tests PT: prothrombin test; INR: international normalized ratio; PTT: partial thromboplastin time; ALT: alanine aminotransferase; ALP: alkaline phosphatase; *: Catheter removal (10 h after the first reintervention); ++: blood analysis before laminectomy; POD1-4: postoperative days 1-4

	Preoperative	POD1	POD2 (1st reintervention)	POD2 *	POD3	POD4 ++
PT (seg)	13.6	17.1	17.1	14.7	15.7	14.8
INR	1.16	1.47	1.47	1.26	1.35	1.27
PTT (seg)	ꟷ	ꟷ	39	ꟷ	36	38
Platelets (G/L)	108	91	87	92	77	87
Fibrinogen (mg/dL)	ꟷ	ꟷ	333	ꟷ	449	517
Albumin (g/L)	45.2	ꟷ	25.5	ꟷ	26.1	ꟷ
ALT (U/L)	69.4	103	82.9	78	68.4	74
ALP (U/L)	155	98	93	92	ꟷ	117
Total bilirubin (mg/dL)	0.7	0.7	0.73	1.15	0.77	0.96

The patient consented to a combination of EA with general anesthesia, and a lower thoracic epidural catheter (T9-T10) was placed without complications.

A two-hour surgery was successfully performed, without the Pringle maneuver and with insignificant blood loss (~100 mL). The patient remained hemodynamically stable, and normothermia was assured. Postoperative monitoring took place in an intermediate care unit, with no complications reported. Prophylactic enoxaparin 40 mg was given 12 hours after the catheter placement and pain relief was obtained with a patient-controlled epidural analgesia (PCEA) device with ropivacaine and sufentanil.

On postoperative day two (POD2), the patient presented with significant blood loss, including extravasation from the surgical drain. An emergent procedure was performed, but no vascular bleeding was found. Therefore, coagulopathy was assumed, and the patient received 1 g of tranexamic acid, one RBC unit, three fresh frozen plasma (FFP) units, and one platelet concentrate (PC) in total. Hemorrhage resolved and following the consultation of coagulation profile (platelet count (PTc) 92 G/L, prothrombin test (PT) 14.7 seg, INR 1.26), and considering a 12-hour interval since the last administration of prophylactic enoxaparin, the epidural catheter was removed.

**Figure 1:**
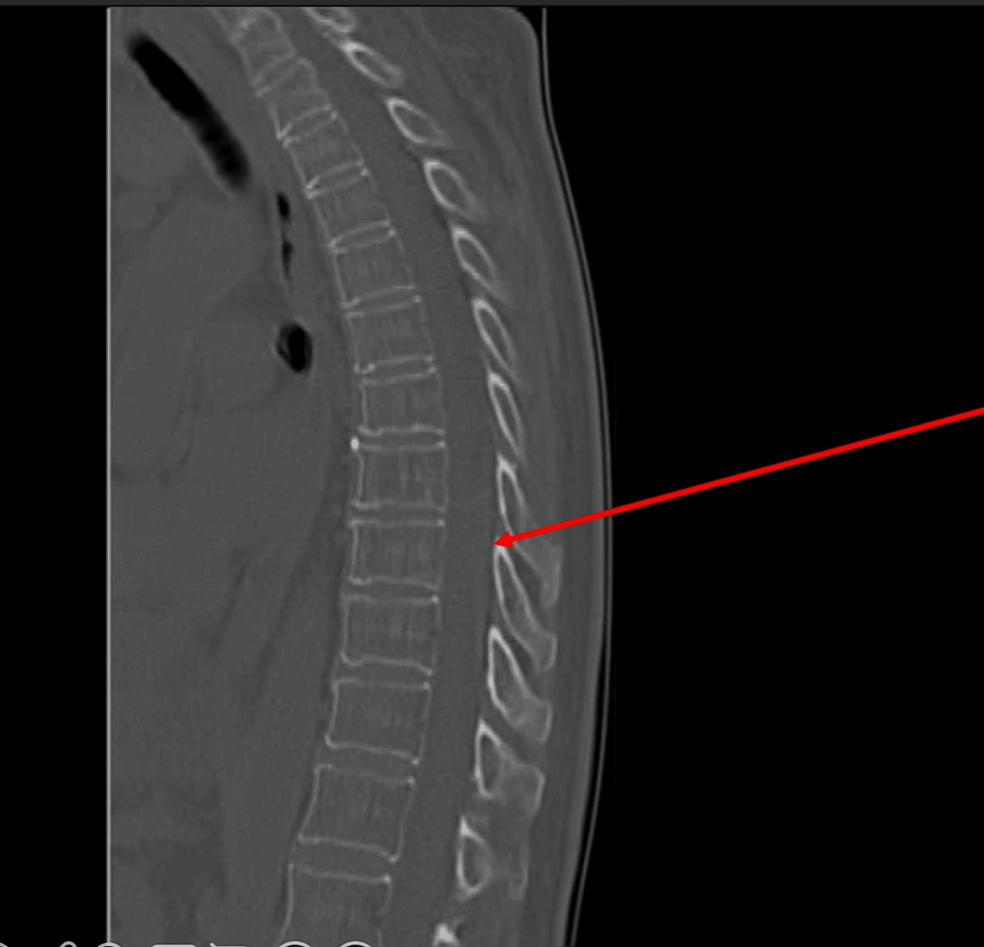
Thoracic and lumbar CT scan A hyperintense lesion displacing the cord anteriorly at T8-T10 (indicated by red arrow).

**Figure 2:**
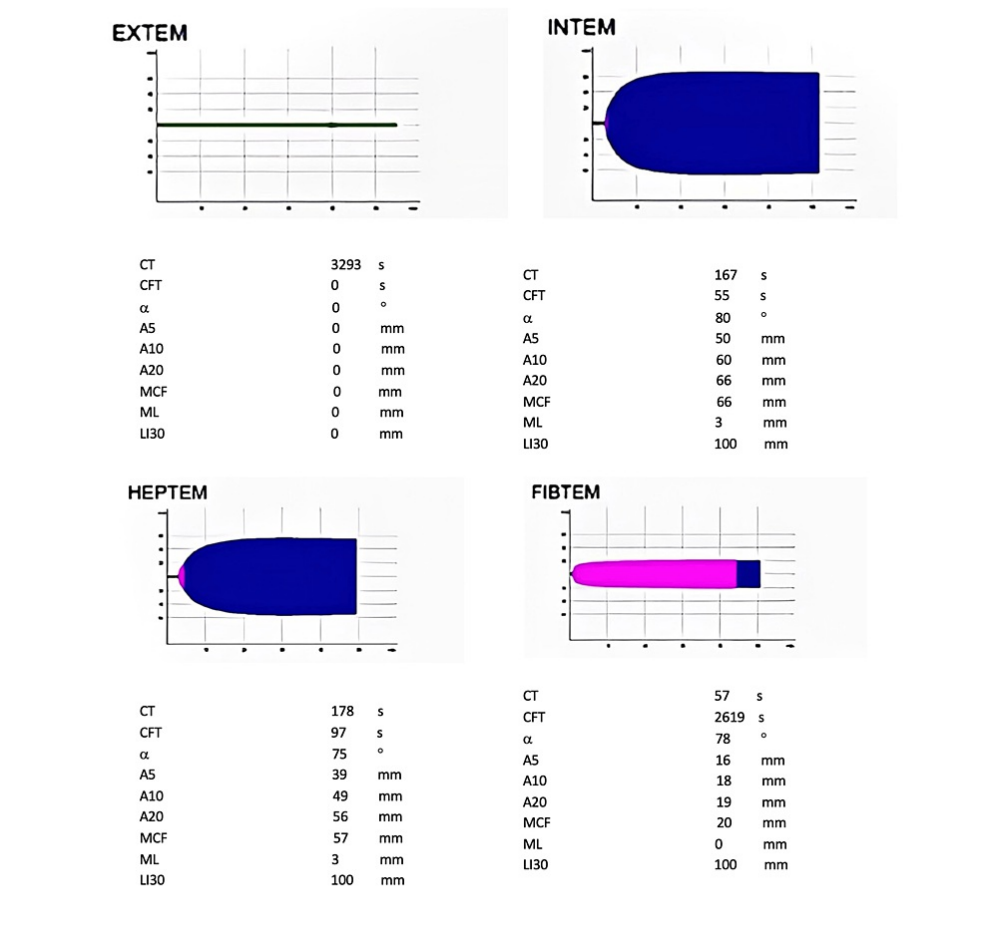
Thromboelastometry showing no activation of clot formation in EXTEM EXTEM: extrinsically activated thromboelastometry

**Figure 3:**
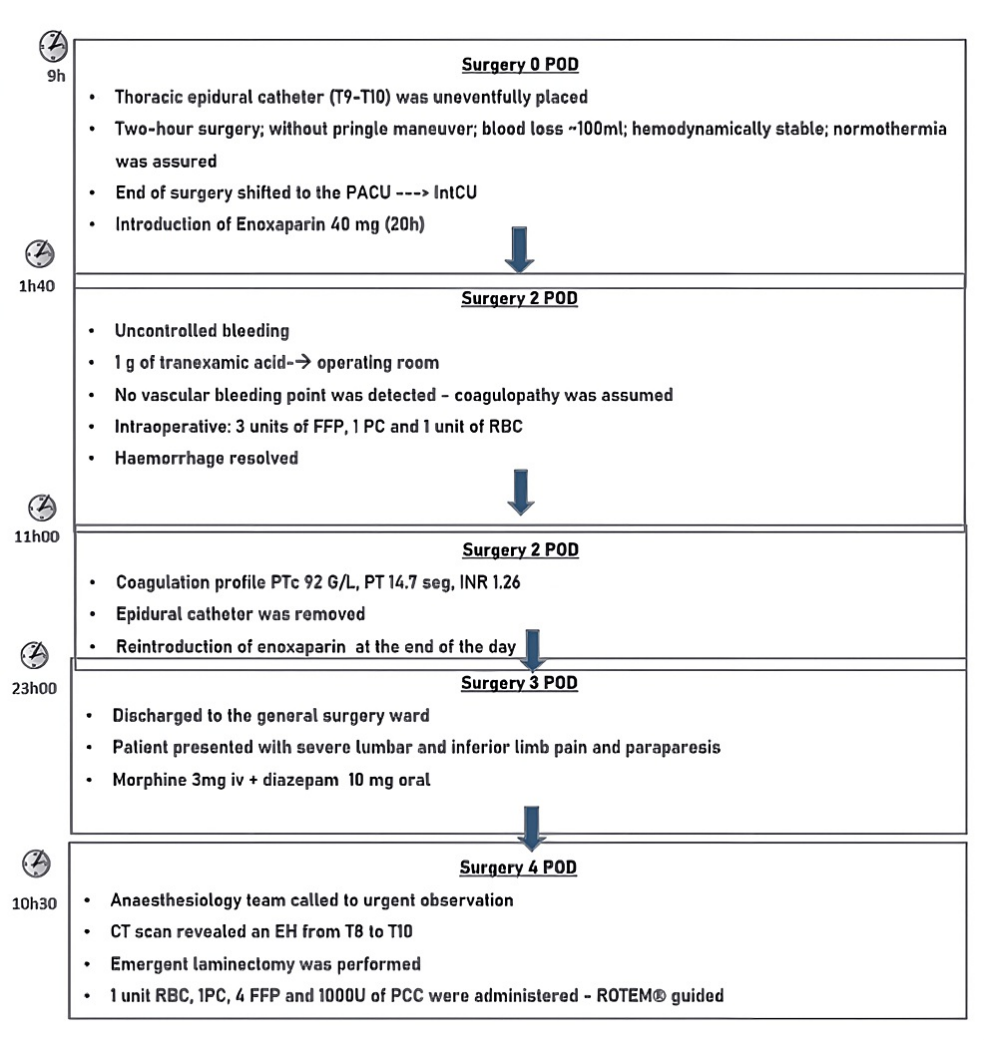
Flowchart of events and the subsequent management PACU: post-anesthesia care unit; IntCU: intermediate care unit; FFP: fresh frozen plasma; PC: platelet concentrate; PCC: prothrombin complex concentrate; PTc: platelet count; PT: prothrombin test; INR: international normalized ratio

The next day, the patient was discharged to the general ward. At the end of the same day, the patient presented with paraparesis plus severe lumbar and inferior limb pain. A CT scan showed an EH from T8 to T10 (Figure 1), and an emergent laminectomy was performed. During the laminectomy procedure, hemostasis was difficult to obtain, and, for that reason, a rotation thromboelastometry (ROTEM ) (Figure 2) was performed. Approximately 1,000 units of prothrombin complex concentrate, four FFPs, one unit RBC, and one PC were administered, ROTEM guided. The sequence of the events mentioned is represented by a flowchart in Figure 3. The postoperative recovery went on without any complication, and since the POD4 when the patient started rehabilitation. The patient managed to walk unassisted after several months of rehabilitation.

## Discussion

Epidural analgesia is considered by many to be the optimal pain relief after major surgery and may improve perioperative outcomes when compared with general anesthesia [4]. Epidural analgesia offers several benefits, such as shorter duration of postoperative ileus, decreased pulmonary complications and diminished stress response, and excellent postoperative analgesia and early ambulation [5].

However, after hepatic resection, coagulopathy can be an issue, despite completely normal preoperative coagulation function, increasing the risk of EH [2]. There are several factors that can contribute to coagulopathic conditions besides liver failure. Factors such as blood loss, acidosis, blood transfusion, and hypothermia [6].

Our case report makes us reflect on some conclusions made in the past concerning the risk of coagulopathy in liver resection. Matot et al. concluded that minor hepatic surgery with an epidural is reasonably safe because only momentary changes in coagulation occurred [7]. Shontz stated that those who developed a hemostatic abnormality were more likely to have had greater estimated blood loss and median volume of liver resected [8]. Greenland has suggested that the main factors contributing to postoperative coagulopathy after liver resection are the quality and functional capability of the residual liver tissue, the presence of cirrhosis, benign or malignant lesions, and the use of the Pringle maneuver and its duration [9]. Recently, Jacquenod et al. [10] in a retrospective cohort study identified five factors independently associated with postoperative coagulation profile disorders: preexisting hepatic cirrhosis, preoperative INR ≥1.3, preoperative PTc <150 G/L, major hepatectomy, and estimated intraoperative blood loss ≥1,000 mL. In our case report, the history of metastatic cholangiocarcinoma and several platelets of 108 G/L were the only identified risks according to these studies.

However, similar to Jacquenod et al.'s findings [10], INR was higher on POD1 and the PTc was lower on PODs 2-3. Additionally, our case confirms previous concerns with catheter removal timing, which accounted for 30-60% of clinical EHs [1].

Nonetheless, our main purpose with this case report is to ponder if INR, activated PTT (aPTT), and PTc give us enough information about security in neuroaxis management in liver resection surgery. Hepatic surgery centers recommend the use of epidural analgesia only in patients with INR <1.5, aPTT <40 seconds, and PTc >80,000/mm. Those are requisites for epidural catheter placement but also for removal [9].

PT-INR has been adopted not only to guide FFP transfusion but also to assist in the decision of when to start thromboembolic prophylaxis following a partial hepatectomy, despite a deficient comprehension of the consequences of a partial hepatectomy on the coagulation cascade [6].

Viscoelastic testing uses whole blood (e.g., involvement of platelets, plasma components of coagulation, RBCs, and WBCs) to evaluate functional coagulation. It measures the rate of formation, stabilization, and lysis of the clot, giving us a more complete picture of the process of coagulation [3].

Notably, if we had requested a ROTEM before removing the epidural catheter our attitudes would have been quite different. The catheter would not be removed in the first place as ROTEM would have shown us the coagulopathy that we had not been able to suspect by standard coagulation tests. Second, we would ask for the collaboration of the transfusion medicine service to enable a more effective targeted hemostatic therapy. We believe if we had requested a ROTEM, the outcome would be different, and patient safety would have improved.

Unfortunately, the evidence available is scant to bring forth definitive answers. By searching PubMed® (keywords: “epidural hematoma” and “epidural hematoma AND liver resection” and “epidural hematoma AND liver surgery”), there is no single case report of EH because of epidural catheter removal or insertion for liver resection surgery. This does not mean that no such cases have occurred, as there is a tendency to underreport adverse outcomes.

It would be interesting to have clinical trials including preoperative and postoperative thromboelastometry in patients undergoing liver resection for a better evaluation of clot formation functionality, actual bleeding risk, and, hence, the appropriateness of epidural analgesia.

Up to now, anesthesiologists depended on neuraxial guidelines concerning coagulation values associated with a safe neuraxial approach even if much of what is written is based on expert opinion [10].

## Conclusions

The use of thoracic epidural analgesia (TEA) in liver surgery must be individualized with steps planned from the beginning. It is crucial to assess the benefits against the risks, analyzing comorbidities, contraindications, coagulation profiles, and hepatic reserve. We suggest that further evaluation of functional coagulation status, rather than laboratory status (e.g., viscoelastic testing, in selected cases, such as those with identified risk factors for coagulopathy) would be of value.
